# Influenza and pneumococcal vaccination in Australian adults: a systematic review of coverage and factors associated with uptake

**DOI:** 10.1186/s12879-016-1820-8

**Published:** 2016-09-26

**Authors:** Amalie Dyda, Surendra Karki, Andrew Hayen, C. Raina MacIntyre, Robert Menzies, Emily Banks, John M. Kaldor, Bette Liu

**Affiliations:** 1 School of Public Health and Community Medicine, University of New South Wales (UNSW), Sydney, New South Wales (NSW) Australia; 2 National Centre for Epidemiology and Population Health, Research School of Population Health, The Australian National University, Acton, Australian Capital Territory Australia; 3 Kirby Institute, UNSW, Sydney, NSW Australia

## Abstract

**Background:**

In the absence of an adult vaccination register, coverage estimates for influenza and pneumococcal vaccination come from surveys and other data sources.

**Methods:**

Systematic review and meta-analysis of studies examining vaccination coverage in Australian adults from 1990 to 2015, focusing on groups funded under the National Immunisation Program, and intervals prior to and following the introduction of universal funding.

**Results:**

Twenty-two studies met the inclusion criteria; 18 used self-report to determine vaccination status. There were 130 unique estimates of coverage extracted. Among adults aged ≥65y, during the period of universal funding (1999-onwards), the summary estimate of annual influenza vaccination coverage from 27 point estimates was 74.8 % (95 % CI 73.4–76.2 %; range 63.9–82.4 %); prior to this period (1992–1998) from 10 point estimates it was 61.3 % (95 % CI 58.0–64.6 %; range 44.3–71.3 %). For the period of universal funding for pneumococcal vaccination (2005-onwards) the summary estimate for coverage was 56.0 % (95 % CI 53.2–58.8 %; range 51.2–72.8 %, 10 point estimates); prior to 2005 it was 35.4 % (95 % CI 18.8–52.0 %; range 15.4–45.2 %). Coverage for both vaccines was significantly higher following the introduction of universal funding. Influenza vaccination coverage in those aged 18–65 years with a medical indication was lower but data were not combined. Seven studies reported on Aboriginal Australians with three studies reporting five coverage estimates for influenza vaccination in adults ≥65 years (range 71 % - 89 %).

**Conclusions:**

Adult influenza and pneumococcal vaccination coverage has increased since the introduction of universal funding, but remains sub-optimal, with pneumococcal coverage lower than influenza. Implications: This review highlights the need for more coverage data overall and in high risk groups, to support public health programs to improve coverage.

## Background

Vaccination is a key public health strategy to mitigate the impact of some important infectious diseases [1].

In Australia, vaccines are funded nationally for children and adults under the Australian National Immunisation Program (NIP), as well being available privately. Over the last 20 years, as new vaccines and evidence supporting their effectiveness have become available, the NIP has undergone a number of changes. For adults, annual influenza vaccination has been funded nationally since 1999 for all those aged ≥65 years and, since 2010, for those less than 65 years of age who are at higher risk of infection, such as people with circulatory and respiratory conditions and pregnant women [2, 3]. All Aboriginal and Torres Strait Islander people aged over 15 years have also been funded for influenza vaccination since 2010. Pneumococcal vaccination has been funded for all adults aged ≥65 years and all Aboriginal and Torres Strait Islander adults aged ≥50 years since 2005 [2]. It is also funded for Aboriginal and Torres Strait Islander adults aged from 15 to 49 years who have medical conditions that increase their risk of invasive pneumococcal disease [4].

An important measure of vaccine program success is coverage. While childhood vaccination coverage can be monitored by the Australian Childhood Immunisation Register [5] and the recently established human papillomavirus (HPV) vaccination program register collects comprehensive data on HPV vaccine provided to adolescents [6], at the time of publication of this review, no national register to document vaccination of older adults existed, although some states have adult registers. Therefore, we conducted a systematic search and analysis of the available evidence to quantify levels of influenza and pneumococcal vaccination coverage reported in adults in Australia, the methods used to measure coverage in these studies, and whether studies reported on other factors associated with influenza and pneumococcal vaccination, such as Aboriginality or medical indications.

## Methods

### Search strategy and terms

This review was developed in line with the PRISMA guidelines [7]. Electronic searches of MEDLINE, the Cochrane Library and EMBASE were conducted to identify relevant journal articles published between 1 Jan 1990 and 31 May 2015. The search terms and strategy used were as follows: “vaccination” or “immunisation” AND “influenza” or “pneumococcal” AND “coverage” or “uptake” or “rate” AND “adult” AND “Australia”. For grey literature, such as government reports, a Google and Google Scholar search was conducted using the search terms; “adult” AND “vaccination” AND “coverage” AND “Australia”. Reference lists of review articles were scanned for relevant publications and experts in the field were also consulted.

All titles and abstracts or executive summaries found through the search strategy were screened (AD) to determine if they were relevant to the review. The full text of those articles that appeared to meet the inclusion criteria were retrieved and reviewed for relevance. Where there was uncertainty the report was reviewed by a co-author.

### Inclusion/Exclusion criteria

Studies were included if they met the following criteria: reported vaccination coverage for influenza, or pneumococcal disease in adults (defined as ≥18 years); were published between January 1990 and May 2015; and involved an Australian population defined only by geographic area and age group. Studies were excluded if they: only described vaccination in a specific population such as healthcare workers, military personnel, pregnant women, and the work place. As a systematic review of pandemic H1N1 influenza vaccination coverage has already been published [8], studies examining this vaccine alone were excluded. No language restrictions were imposed.

### Data extraction and synthesis

A standard data extraction form was used and two authors (AD and SK) independently extracted data. The data extracted from each study included: article ID; study design including sampling strategy; sample size; time period; study population (e.g. location, age); method of vaccination status ascertainment, reported influenza and pneumococcal vaccination coverage, any factors reported to be associated with vaccination. Where it was missing or unclear, authors or organisations were contacted to obtain additional data.

Data extracted from each study were tabulated. They were then grouped according to the infection the vaccine prevented (seasonal influenza strains or S pneumoniae) and also by age group (≥65 years, <65 years). As the lower age bound sampled varied between studies, for convenience, the younger adult age group is referred to as <65 years. Data from studies reporting influenza coverage specifically in adults aged <65 years with risk factors for complications and serious disease following influenza infection (i.e. those funded for influenza vaccination from 2010) was shown separately. Also studies reporting influenza and/or pneumococcal vaccination specifically in Aboriginal and Torres Strait Islander adults were shown separately.

For estimates of vaccination coverage, to preserve individual survey sampling designs, we used the point estimates and the standard error of the proportion provided by the study. Where 95 % confidence intervals (CIs) but not standard errors were reported, we calculated the standard error from the CIs. Where neither were provided, we calculated the standard error using the standard formula for the standard error for a proportion using the raw data (i.e. the numerator and denominator).

### Meta-analyses

A random effects meta-analysis using the inverse variance method [9] was conducted to provide an overall estimate of coverage where seven or more data points were available for the same vaccine type, age group and time period (i.e. the time period subsequent to the introduction of universal funding for that vaccine in the population group under examination). Estimates of coverage and summary estimates for studies included in the meta-analyses were then presented in forest plots for each vaccine. The results were reported as vaccinated proportion with 95 % confidence intervals. Analyses were conducted using STATA version 12.1.

### Ethics

This study was approved by the University of New South Wales Human Research Ethics Panel (Social/Health Research) (9-14-12).

## Results

**Fig. 1:**
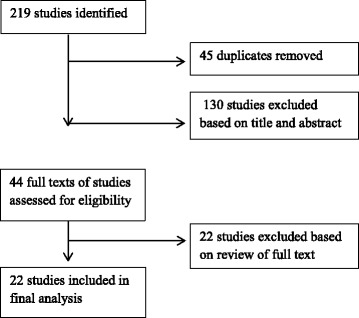
Flow diagram of search strategy results- description. This figure shows the number of studies included and excluded at each stage of the review process

The initial search strategy retrieved a total of 219 titles and abstracts, of which 45 were duplicates. After screening the titles and abstracts, the full texts of 44 reports were retrieved of which 22 (including 15 peer-reviewed and seven non-peer reviewed reports) met the inclusion criteria for the review (see Fig. 1). Of those reports retrieved but not included, ten studies did not report vaccination coverage, four studies investigated an intervention and did not report coverage, four reported coverage only for a specific population sub-group, three reported only on vaccine efficacy or safety, and one paper reported on a subset of data from one of the reports included.

### Summary of included studies

Table 1 provides a summary of the characteristics of each included study. Twenty-one studies were cross-sectional and one was a case-cohort design [10]. The number of participants ranged from 50 to 15836 (median = 5590, interquartile range (IQR) 1462 to 10231). The age ranges sampled varied considerably. Twelve studies collected data from people aged ≥18 years, five from people aged ≥15 years (one of these included children as well), two from people aged ≥40 years, two from people aged ≥65 years and one from people aged ≥60 years. Within each study, vaccination coverage was reported for a variety of different age groups and some studies, such as the NSW Ministry of Health reports [11], included separate measures of coverage for many individual years in unique population samples. Hence there were substantially more point estimates of vaccination coverage than the number of included studies. Coverage estimates in Tables (Tables 2, 3, and 4) are therefore listed by both the study name and the year of reported coverage.

**Table 1:** Summary of included studies

Article ID	Vaccine type	Time period study conducted	Year coverage reported for	Participants age (years)	Sample size	Study design	Sampling frame	Data collection method
MacIntyre R, 1993 [25]	Influenza	May–June 1992	1992	0+	537	Cross sectional	Systematic sampling from Victorian White Pages	Telephone interviews; self-report
Hanna, 2001 [23]	Influenza and pneumococcal	1995–2000	Influenza 1995–2000	15+	15836	Cross sectional	All data for Indigenous adults extracted for Far North Queensland region from health department database (VIVAS database)	Provider reported to health department database
			Pneumococcal 1995–2000					
Taylor, 2001 [3]	Influenza and pneumococcal	1998–2000	Influenza annually 1998, 1999, 2000	18+	10505	Cross sectional	Systematic sampling from the Australia wide White Pages	Telephone interviews; self-report
			Pneumococcal 1996–2000					
Andrews, 2005_V [26]	Pneumococcal	July–Aug 2000	1999 and 2000	65+	278	Cross sectional	Participants from the 1999 Victorian Population Health Survey who agreed to be contacted for further studies were sampled. Contacted by phone for permission and details before contacting provider	Telephone interviews; provider report
Gill, 2007 [30]	Influenza	1993–2004	Reported annually for each year of study	15+	8448	Cross sectional	Not reported	Telephone interviews; self-report
Horby, 2005 [19]	Influenza	Oct–Nov 2001	2001	40+	7681	Cross sectional	RDD* from Australia wide White Pages	Telephone interviews; self-report
Hanna, 2003 [24]	Influenza	2001	2002	15+	11062	Cross sectional	All data for Indigenous adults extracted for Far North Queensland region from health department database (VIVAS database)	Provider reported to health department database
Menzies, 2004 [21]	Influenza and pneumococcal	2001	Influenza 2001 Pneumococcal 1997–2001	18+	3681	Cross sectional	National Health Survey- 3 stage random community sample –Australia wide- for Indigenous adults	Face to face interviews; self-report
Andrews, 2005 [10]	Influenza and pneumococcal	Apr 2000–Mar 2002	Influenza 2000 and 2001	65+	2934	Case cohort	Random sample from 2 Victorian hospitals. End of each month participants randomly selected from those discharged in that month.	Telephone interviews; provider report
			Pneumococcal 2000 and 2001					
NSW Health, 1997–2012 [11]	Influenza and pneumococcal	2002–2012	Reported each year of study.	18+	3416	Cross sectional	RDD* from NSW White pages- mobile number’s included from 2012	Telephone interviews; self-report
			Influenza annually					
			Pneumococcal <5 years					
AIHW, 2003 [12]	Influenza	2002	Influenza 2002 Pneumococcal 1998–2002	40+	8000	Cross sectional	RDD*, Australia wide	Telephone interviews; self-report
Hanna, 2004 [22]	Influenza	2003	2003	15+	11204	Cross sectional	All data for Indigenous adults extracted for Far North Queensland region from health department database (VIVAS database)	Provider reported to health department database
AIHW, 2005 [13]	Influenza and pneumococcal	2004	Influenza 2004 Pneumococcal 2000–2004	18+	7500	Cross sectional	RDD*, Australia wide	Telephone interviews
Menzies, 2008 [20]	Influenza and pneumococcal	2004–2005	Influenza 2004/2005	18+	10439	Cross sectional	RDD*, Australia wide for Indigenous adults	Face to face interviews
			Pneumococcal 2001–2005					
AIHW, 2008 [14]	Influenza and pneumococcal	2006	Influenza 2006 Pneumococcal 2002–2006	18+	8022	Cross sectional	RDD*, Australia wide	Telephone interviews; self-report
Ridda, 2008 [28]	Influenza and pneumococcal	June-Nov 2006	Reported ever vaccinated previously	60+	200	Cross sectional	Convenience sampling of consecutive inpatients from one Sydney hospital- validated with medical records-	Face to face interview; self-report
Dower, 2011 [27]	Influenza and pneumococcal	2008	Influenza 2008	18+	2203	Cross sectional	RDD*, Queensland	Telephone interviews; self-report
			Pneumococcal 2004–2008					
AIHW, 2011 [15]	Influenza and pneumococcal	2009	Influenza 2009 Pneumococcal 2005–2009	18+	10231	Cross sectional	RDD*, Australia wide	Telephone interviews; self-report
Loke, 2012 [29]	Influenza and pneumococcal	April–May 2011	Influenza annually 2010, 2011	18+	50	Cross sectional	Convenience sampling of random selection of inpatients from a South Australian hospital	Face to face interview; self-report
			Pneumococcal ever vaccinated					
BEACH 2014 [16]	Pneumococcal	2013	2009–2013	15+	2523	Cross sectional	Random sample of 125 GP practices- Australia wide –Sample initially source all GPs who claimed a min 375 GP A1 Medicare items. Randomly selected GPs are then approached by letter then phone.	Provider reported from GP records
Cheng, 2013 [17]	Influenza	2012	2012	18+	1216	Cross sectional	National hospital based sentinel surveillance- Australia wide. Hospitals recruited from physician members of the Thoracic Society of Australia and New Zealand Pandemic (H1N1) 2009 Task Force and representatives of the Australasian Society for Infectious Diseases. Also recruited 2 non-urban hospitals to increase sample of Indigenous patients.	Standard collection instrument, collected clinical, radiological and laboratory data- self report, or medical record (generally based on self-report)
Cheng, 2014 [18]	Influenza	2013	2013	18+	964	Cross sectional	National hospital based sentinel surveillance- Australia wide. Hospitals recruited from physician members of the Thoracic Society of Australia and New Zealand Pandemic (H1N1) 2009 Task Force and representatives of the Australasian Society for Infectious Diseases. Also recruited 2 non-urban hospitals to increase sample of Indigenous patients.	Standard collection instrument, collected clinical, radiological and laboratory data- self report, or medical record (generally based on self-report)

*RDD random digit dialling

**Table 2:** Annual influenza vaccination coverage by age group and year

Study ID	Year influenza vaccine given	Estimated coverage (%)	Number vaccinated/Total population
Age ≥65 years			
MacIntyre 1993 [25]	1992	44.4	43/97
Gill 2007 [30]	1993	55.6	265/477
Gill 2007 [30]	1994	55.5	282/508
Gill 2007 [30]	1995	62.3	320/514
Gill 2007 [30]	1996	67.1	349/520
Gill 2007 [30]	1997	67.7	773/1141
NSW Ministry of Health 1997 [11]	1997	56.0	1840/3283
Gill 2007 [30]	1998	71.3	829/1163
Taylor, 2001 [3]	1998	60.7	4389/7228
NSW Ministry of Health 1998 [11]	1998	63.8	2169/3401
Gill 2007 [30]	1999	77.1	897/1163
Taylor, 2001 [3]	1999	69.8	5047/7228
Andrews, 2005_V [26]	1999	72.3	N/A
Gill 2007 [30]	2000	80.3	846/1054
Taylor, 2001 [3]	2000	74.1	5357/7228
Andrews, 2005 [10]	2000	78.4	N/A
Gill 2007 [30]	2001	79.8	451/565
Horby 2005 [19]	2001	78	N/A
Andrews, 2005 [10]	2000	67.4	1978^a^/2934
Andrews, 2005 [10]	2001	74.5	2186^a^/2934
NSW Ministry of Health 2002 [11]	2002	75.6	2549/3419
AIHW 2003 [12]	2002	76.9	NA
Gill 2007 [30]	2002	80.1	306/382
NSW Ministry of Health 2003 [11]	2003	75.5	2692/3577
Gill 2007 [30]	2003	81.2	310/382
Gill 2007 [30]	2004	82.4	477/579
AIHW 2005 [13]	2004	79.1	4067^a^/5141
NSW Ministry of Health 2004 [11]	2004	75.4	2022/2706
NSW Ministry of Health 2005 [11]	2005	74.7	2508/3390
NSW Ministry of Health 2006 [11]	2006	75.1	1744/2388
AIHW 2008 [14]	2006	77.5	4356^a^/5621
NSW Ministry of Health 2007 [11]	2007	72.3	1698/2347
NSW Ministry of Health 2008 [11]	2008	71.4	1935/2742
NSW Ministry of Health 2009 [11]	2009	72.3	2537/3561
AIHW, 2011 [15]	2009	74.6	3959^a^/5307
NSW Ministry of Health 2010 [11]	2010	72.6	2559/3601
NSW Ministry of Health 2011 [11]	2011	72.4	3470/4749
Loke, 2012 [22]	2011	63.9	23/36
NSW Ministry of Health 2012 [11]	2012	68.4	2799/4030
Cheng 2013 [17]	2012	77.6	420/541
Cheng 2014 [18]	2013	80.5	178/221
Age <65 years			
Horby, 2005 [19]	2001	24.0	N/A
AIHW 2003 [12]	2002	18.6	N/A
AIHW 2005 [13]	2004	18.8	N/A
AIHW 2008 [14]	2006	20.2	485^a^/2401
Ridda, 2007 [17]	2006	63.6	124/195^a^
AIHW, 2011 [15]	2009	22.8	3200/14035^a^
AIHW, 2011 [15]	2009	36.2	NA
Loke, 2012 [29]	2011	21.4	3/14
Cheng 2013 [17]	2012	40.0	270/675
Cheng 2014 [18]	2013	49.2	92/187
Age ≥65 years with risk factors			
Dower, 2011 [27]	2008	81.7	594/727^a^
Cheng 2013 [17]	2012	78.5	397/506
Cheng 2014 [18]	2013	80.4	172/214
Age <65 years with risk factors			
Taylor 2001 [3]	2000	32.2	367/NA
Horby 2005 [19]	2001	34.0	N/A
AIHW 2003 [12]	2002	41.7	N/A
AIHW 2005 [13]	2004	41.6	N/A
AIHW 2008 [14]	2006	29.8	N/A
Dower, 2011 [27]	2008	46.5	345/742^a^
AIHW, 2011 [15]	2009	36.2	N/A
Cheng 2013 [17]	2012	44.7	238/532
Cheng 2014 [18]	2013	49.2	92/187

*N/A* not available; data not available from estimates provided or contacting authors

^a^raw numbers calculated by authors as not provided in study

**Table 3:** Pneumococcal vaccination by age group and year

Study ID	Year/s pneumococcal vaccine given	Estimated coverage (%)	Number vaccinated/Total population
Age ≥65 years			
Andrews, 2005 [10]	1996–2000	57.9	N/A
Taylor, 2001 [3]	1996–2000	15.4	991/6435^a^
Andrews, 2005 [10]	1997–2001	49.1	NA
Andrews, 2005 [10]	1998–2002	56.3	NA
NSW Ministry of Health 2002 [11]	1998–2002	37.8	1294/3419
NSW Ministry of Health 2003 [11]	1999–2003	45.2	1617/3577
NSW Ministry of Health 2004 [11]	2000–2004	43.2	1169/2706
AIHW 2005 [13]	2000–2004	51.1	N/A
NSW Ministry of Health 2005 [11]	2001–2005	51.9	1759/3390
NSW Ministry of Health 2006 [11]	2002–2006	58.0	1385/2388
Ridda, 2007 [28]	2006	50.3	98/195^a^
AIHW 2008 [14]	2002–2006	62.2	NA
NSW Ministry of Health 2007 [11]	2003–2007	56.8	1332/2347
NSW Ministry of Health 2008 [11]	2004–2008	56.2	1541/2742
Dower, 2011 [27]	2008	67.6	498/737
NSW Ministry of Health 2009 [11]	2005–2009	53.4	1900/3561
AIHW 2011 [15]	2005–2009	54.4	2679^a^/4924
NSW Ministry of Health 2010 [11]	2006–2010	51.2	1844/3601
NSW Ministry of Health 2011 [11]	2007–2011	56.1	2664/4749
Loke 2012 [29]	2011	54.1	20/37
NSW Ministry of Health 2012 [11]	2008–2012	50.5	2036/4030
BEACH 2014 [16]	2009–2013	72.8	685^a^/941
Age <65 years			
NSW Ministry of Health 2002 [11]	1998–2002	6.4	229/3501
NSW Ministry of Health 2003 [11]	1999–2003	6.6	236/3568
NSW Ministry of Health 2004 [11]	2000–2004	8.0	227/2838
NSW Ministry of Health 2005 [11]	2001–2005	9.2	313/3403
AIHW 2008 [14]	2002–2006	6.5	N/A
NSW Ministry of Health 2006 [11]	2002–2006	10.3	246/2381
NSW Ministry of Health 2007 [11]	2003–2007	10.3	243/2348
NSW Ministry of Health 2008 [11]	2004–2008	10.8	296/2731
Dower 2011 [27]	2008	25.6	181/707^a^
Dower 2011 [27]	2008	11.04	58/525^a^
AIHW 2011 [15]	2005–2009	4.8	258^a^/5307
NSW Ministry of Health 2009 [11]	2005–2009	10.1	351/3489
NSW Ministry of Health 2010 [11]	2006–2010	11.1	383/3458
Loke 2012 [29]	2011	23.1	3/13
NSW Ministry of Health 2011 [11]	2007–2011	13.4	588/4399
NSW Ministry of Health 2012 [11]	2008–2012	10.9	448/4126
BEACH 2014 [16]	2009–2013	33.9	^a^809/2386
Age <65 years with risk factors			
Taylor 2001 [3]	2000	3.5	39/1114^a^
BEACH 2014 [16]	2009–2013	57.9	770/1330

*N/A* not available

^a^raw numbers calculated by authors as not provided in study

**Table 4:** Influenza and/or pneumococcal vaccination in Aboriginal and/or Torres Strait Islander Australians by age range and time period

Disease	Study ID	Year/s vaccination given	Age range	Estimated coverage (%)	Number vaccinated/Total population
Influenza					
	Hanna, 2003 [24]	2001	15–49	85	7719/9081
	Hanna, 2004 [22]	2003	15–49	85	7671/9025
	Menzies, 2008 [20]	2004–2005	18–49	23	N/A
	AIHW 2011 [15]	2009	≥18	27.5	N/A
	Taylor, 2001 [3]	1998	40-64*	9.4	1/10
	Taylor, 2001 [3]	1999	40-64*	19.9	3/15
	Taylor, 2001 [3]	2000	40-64*	22.5	3/13
	Hanna, 2004 [22]	1995–2000	≥50	96	2582/2690
	Hanna, 2003 [24]	2001	≥50	59.4	3275/5513
	Menzies 2004 [21]	2001	≥50	51	N/A
	Hanna, 2004 [22]	2003	≥50	63	3533/5608
	Menzies 2004 [21]	2001	50–64	47	N/A
	Menzies, 2008 [20]	2004–2005	50–64	52	N/A
	Taylor, 2001 [3]	1998	≥65	80.1	19/24
	Taylor, 2001 [3]	1999	≥65	89.0	21/24
	Taylor, 2001 [3]	2000	≥65	78.2	19/24
	Menzies 2004 [21]	2001	≥65	71	N/A
	Menzies, 2008 [20]	2004–2005	≥65	84	N/A
Pneumococcal					
	Hanna, 2001 [23]	1995–2000	15–49	73	3274/NA
	Menzies, 2008 [20]	2001–2005	18–49	12	N/A
	Hanna, 2001 [24]	1995–2000	≥50	73	1971/2700
	Menzies 2004 [21]	2001	≥50	25	N/A
	Menzies 2004 [21]	2001	50–64	20	N/A
	Menzies, 2008 [20]	2001–2005	50–64	30	N/A
	Menzies 2004 [21]	2001	≥65	47	N/A
	Menzies, 2008 [20]	2001–2005	≥65	48	N/A

*N/A* not available

Of the 22 studies, 11 sampled Australia-wide (one of which looked specifically at the Aboriginal and Torres Strait Islander population) [3, 12–21], three focused specifically on Aboriginal and Torres Strait Islander adults in far north Queensland [22–24], three focused on Victoria [10, 25, 26], the remaining two focused on NSW [11] and Queensland [27] respectively and two were based on estimates from a single hospital [10, 28, 29]. The most common (*n* = 10; [3, 11–15, 19, 25, 27, 30]) methodology was to ascertain vaccination coverage using self-reported vaccination status obtained through interviews conducted after random-digit dialling potential participants; 4 smaller studies used self-report from face-to-face interviews and 5 used provider reported vaccination status [10, 16–18, 26]. Three studies described coverage using records extracted from state-based vaccination databases, which rely on health care providers reporting vaccination to the register [22–24]. Seven studies reported on vaccination coverage specifically in Aboriginal and Torres Strait Islander people [15, 20–24]. For 16 studies the primary aim was to obtain information on vaccination [3, 10, 12–15, 19, 22–30] whilst for six others, data on vaccination was collected as part of a larger health survey [11, 16–18, 20, 21].

### Influenza vaccination coverage

A total of 18 studies identified reported annual influenza vaccination coverage in the general adult population aged ≥18 years (see Table 2), with 16 (88.9 %) including coverage data specifically in those aged ≥65 years. From these studies a total of 63 point estimates covering years from 1992 to 2013 were extracted.

Estimated coverage in those aged ≥65 years ranged from 44.4 to 82.4 % over the whole period examined, 1990–2015 (Table 2). Reported coverage prior to the introduction of funded vaccination in 1999 ranged from 44.4 to 71.3 % with coverage following 1999 ranging from 63.9 to 82.4 %. Coverage following 1999 was significantly higher than that prior to 1999 (*p* > 0.001); the summary coverage estimate prior to 1999 from 10 data points was 61.3 % (95 % CI 58.0–64.6 %); versus 74.8 % (95 % CI 73.4–76.2 %); from 1999 onwards from 27 data points.

**Fig. 2:**
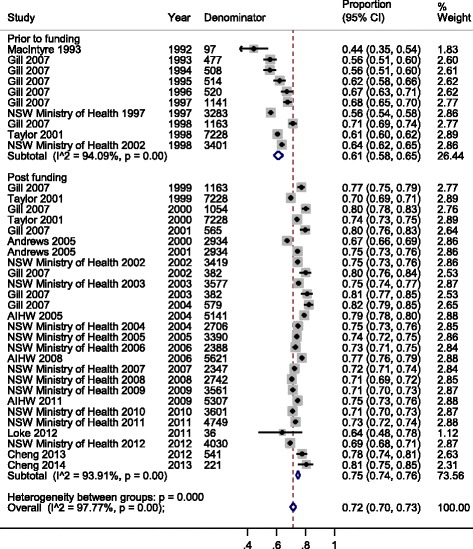
Meta-analysis of eligible studies of influenza vaccination coverage, before and after the introduction of universal funding of vaccine for adults aged ≥65 years. This figure shows the results from the meta-analysis of studies reporting influenza vaccination coverage, before and after the introduction of universal funding of vaccine for adults aged ≥65 years

There was significant heterogeneity in the estimates of influenza vaccine coverage in those aged ≥65 years from 1999 onwards (I2 = 94.1 %, *p* < 0.001) and also among the estimates prior to 1999 (I2 = 93.9 %, *p* < 0.001) ((Fig. 2). To explore this in the data collected from 1999 onwards, we examined the data in subgroups based on location (NSW or Australia-wide; Appendix). For data collected in NSW (11 data points) the summary estimate of coverage was 73.5 % (95 % CI 72–74 %); I2 = 94.0 %, *p* < 0.001 while for data collected Australia-wide (7 data points) the summary estimate of coverage was 76.4 % (95 % CI 73–79 %); I2 = 96.6 %, *p* < 0.001. The test for heterogeneity between NSW and Australia-wide estimates was significant; *p* = 0.03.

There were 9 studies which reported influenza vaccination coverage specifically in people identified as having a chronic disease that made them more susceptible to influenza and its complications [3, 12–15, 17–19, 27] (Table 2). Between 2000 and 2013, vaccination coverage estimates for adults aged <65 years with chronic diseases ranged from 29.8 to 49.2 %; after 2010 when funding was introduced to target this group, two studies estimated vaccination coverage of 44.7 and 49.2 %. In adults aged ≥65 years with chronic diseases, coverage was substantially higher, ranging from 78.5 to 81.7 %.

### Pneumococcal vaccine coverage

Eleven studies were identified that reported pneumococcal vaccination coverage in adults aged ≥18 years (Table 3). From these studies a total of 41 point estimates covering years from 1996 to 2013 were extracted. Among studies reporting vaccination coverage in those aged ≥65 years, estimates ranged from 15.4 to 72.8 %. Among studies reporting vaccination coverage for those aged <65 years, estimates ranged from 4.8 to 33.9 % but the age ranges sampled were inconsistent with some reporting from adults aged ≥18 and some from age ≥50 years. There were also differences in the time period for reported pneumococcal vaccination, with some studies reporting pneumococcal vaccine received within the previous year and others reporting within the previous 5 years (see Table 3).

**Fig. 3:**
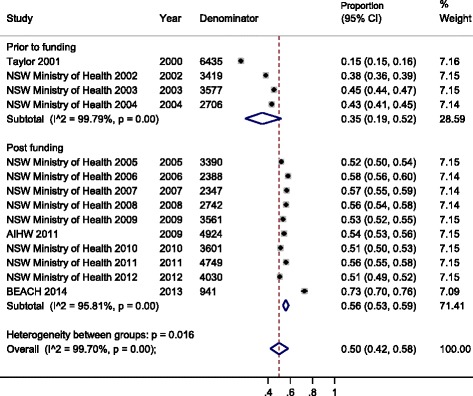
Meta-analysis of eligible studies of pneumococcal vaccination coverage before and after the introduction of universal funding of vaccine for adults aged ≥65 years. This figure shows the results from the meta-analysis of studies reporting pneumococcal vaccination coverage, before and after the introduction of universal funding of vaccine for adults aged ≥65 years

Among adults aged ≥65 years, in 5 studies conducted prior to 2005 [3, 10, 11, 13, 26], the proportion reporting pneumococcal vaccination in the last 5 years ranged from 15.4 to 57.9 %; in 7 studies conducted from 2005 onwards it ranged from 50.3 to 72.8 % [11, 14–16, 27–29]. Coverage from 2005 onwards was significantly (*p* = 0.02) higher than that prior to 2005; the summary coverage estimate prior to 2005 from 4 data points was 35.4 % (95 % CI 18.8–52.0 %) versus 56.0 % (95 % CI 53.2 %–58.8 %) from 2005 (10 data points) (Fig. 3). Eight of the data points for pneumococcal coverage after 2005 came from one study [11]. As with influenza, there was significant heterogeneity in the summary estimates of coverage both prior and after universal funding (*p* < 0.001 for both; Fig. 3).

### Influenza and pneumococcal vaccine coverage in Aboriginal and Torres Strait Islander Australians

Seven studies reported influenza and/or pneumococcal vaccination coverage in Aboriginal and Torres Strait Islander adults [15, 20–24] (Table 4). The recommendations and funding for influenza vaccination of Aboriginal adults were extended to include all those aged ≥15 years in 2010. All studies that met our inclusion criteria were conducted prior to 2010. Sample sizes ranged from 10 to 15836 with coverage reported for a number of different age groups. Four of the studies were Australia-wide, while the other three were based in far north Queensland. Among studies reporting influenza vaccination coverage, two studies estimated coverage of 85 % in those aged 15–49 years [22, 24], one study estimated coverage of 23 % in those aged 18–49 years [20], one study estimated coverage of 27.5 % in those aged ≥18 years [15]. In those aged ≥50 years coverage estimates ranged from 51 to 96 % (4 estimates) [21, 22, 24] and in those aged ≥65 years the available estimates ranged from 71 % to 89 % [20, 21]. For pneumococcal vaccination coverage, 2 estimates for those aged 50–64 years were available, reporting 20 and 30 %. Similarly, there were 2 pneumococcal estimates available for those aged ≥65 years, reporting 47 and 48 % [20, 21].

### Factors associated with receipt of vaccination

Seven studies reported on individual characteristics and other factors associated with receiving vaccination among adults [3, 19, 25, 27–30]. The majority reported on factors associated with influenza vaccination [3, 19, 25, 29, 30], however two studies reported factors associated with both influenza and pneumococcal vaccination [27, 28]. All studies used a cross-sectional design and an interviewer-administered questionnaire (either by phone or in person).

The most important predictor of vaccination was being aged ≥65 versus <65 years, with a statistically significant association found in five studies [3, 25, 27–29]. All other studies that reported influenza vaccination by age showed a higher point estimate of coverage in the older age group although no statistical tests were described.

After adjusting for age, a number of factors were consistently reported to be significantly associated with vaccination. Two studies reported a statistically significant association between lower education (versus higher education level) and greater likelihood of vaccination with adjusted odds ratios ranging from 1.12 (95 % CI 1.01–1.21) to 1.5 (95 % CI 1.1–1.9) [3, 19]. Two studies reported statistically significant associations between lower income (versus higher income) and vaccination with adjusted odds ratios ranging from 1.4 (95 % CI 1.1–2.3) to 2.96 (95 % CI 1.59–5.52) (3, 19). Having a medical risk factor, being female, living in a metropolitan area, having a positive perception of vaccination, doctor recommending influenza vaccination to the patient, having poor self-reported health status and believing the vaccine to be effective were each reported to be significantly associated with greater likelihood of vaccination in two studies each [3, 19, 25]. Being a non-smoker [27], believing the vaccine will not make you ill, having more correct knowledge [28] and having a positive attitude towards vaccination [28] were all found to be significantly associated with vaccination in one study each.

In two studies of Aboriginal and Torres Strait Islander adults, being aged ≥50 versus those aged 18–49 and living in a remote versus non-remote area were consistently reported as having greater likelihood of being vaccinated for influenza and pneumococcal disease although no statistical tests were presented [20, 21].

## Discussion

In this review we found 22 studies yielding 81 data points on influenza and 49 data points on pneumococcal vaccination coverage between 1990 and 2015 for a general population of adults in Australia. Studies in relation to influenza vaccination ranged over a wider time period than those for pneumococcal vaccination. The majority of included studies focused on annual influenza vaccination coverage and used a cross sectional study design. Random-digit dialling of potential participants and self-reported vaccination status obtained through interviews were the commonest methods employed to ascertain data. A small number of studies collected data from surveying healthcare providers directly about their patients or from a state based immunisation database, which requires providers to report immunisations given. Most studies had sample sizes of over 1000 participants.

Around three-quarters of adults aged ≥65 years were estimated to have received annual influenza vaccination in the period after 1999. Vaccination coverage was higher following the introduction of the funded universal vaccination program for people aged ≥65 years. The current national immunisation strategy considers the influenza vaccination coverage in people aged ≥65 years to be relatively low and suggests coverage needs to improve. However, there are no official national coverage targets for adults, and no clear plan for improving coverage [31].

Coverage estimates were lower in people aged <65 years with medical risk factors surveyed after 2010, who were also funded to receive influenza vaccine, compared to coverage in people aged ≥65 years with risk factors. While there were only 2 studies that reported on this period and the sample sizes were relatively small [17, 18], this finding suggests that factors other than funding are important for improving vaccination coverage in adults. Universal recommendations have historically been more successful with achieving high coverage than targeted recommendations for risk groups [32], and this is supported by our findings. This may be due to the fact that universal recommendations are usually easier to implement.

Pneumococcal vaccination coverage also appeared to be influenced by funding, with a summary estimate of coverage in the ≥65 year age group prior to the introduction of funding in 2005 of 35.4 % (95 % CI 18.8 %–52.0 %). However even after 2005, coverage estimates for pneumococcal were lower than that for influenza at around 56 %. This difference may arise due to a greater awareness of the availability and need for influenza vaccine. It could also be that pneumococcal coverage levels are under-reported. Only one of the identified studies of pneumococcal coverage in ≥65 year olds (the ‘Bettering the evaluation and care of health’ (BEACH) study [16]) relied on provider report, as opposed to self- reported vaccination status. Coverage reported from this study was substantially higher at 72.8 % and more comparable to that for influenza in ≥65 year olds. Recall bias may affect both self-report for pneumococcal and influenza vaccination, but could possibly be less for influenza as it is given annually. The higher coverage reported in the BEACH study may also have been affected by the sample, which is not population-based, and only includes people who attend GPs.

All of the available data on influenza and pneumococcal vaccination coverage in Aboriginal and Torres Strait Islander adults comes from studies that were conducted prior to 2010 when funding for influenza vaccination of Aboriginal and Torres Strait Islander adults was limited to adults aged ≥50 years and adults aged <50 years with risk factors [4]. Influenza vaccination coverage reported in Aboriginal and Torres Strait Islander adults aged ≥65 years appeared to be similar to the general Australian adult population of the same age. Two of the three studies which reported on influenza vaccination coverage in Aboriginal and Torres Strait Islander adults for this age group had large samples of Aboriginal and Torres Strait Islander adults and included sampling and recruitment in remote areas, making it likely the findings are robust and generalisable. Lack of data in consistent age groups or time periods makes it difficult to make comparisons of pneumococcal vaccination coverage in Aboriginal and Torres Strait Islanders and the general Australian adult population.

In both meta-analyses of influenza and pneumococcal vaccination coverage in ≥65 year olds we found significant heterogeneity. The large sample sizes of most studies is likely to have contributed to this, by making small differences in coverage statistically significant. Despite this, we attempted to explore possible reasons for this heterogeneity. When data for NSW influenza coverage among adults were combined and compared to a combined estimate for Australia-wide coverage we found a significantly higher estimate for national coverage; it is unclear what the reasons may be for this. Another possible source of heterogeneity was differences in data collection methods (e.g. self-report vs provider report vs state based databases), however there were too few data points from studies using provider report and state-based databases to make reliable comparisons. The most effective and accurate methods of data collection requires further exploration, particularly to help inform the development of the recently announced whole-of-life vaccination register in Australia.

Comparing coverage reported in this systematic review for Australia to other high income countries with funded vaccination programs, there were some similarities and differences. Data from England for 2014/2015 showed influenza vaccination coverage of 72.7 % in those aged ≥65 years [33]. For the same time period influenza vaccination coverage in those aged <65 years with risk factors appeared to be slightly higher in England (50.3 %) [33] compared to the estimates we found for Australia. In both countries both groups are funded for free influenza vaccination [33]. In the United States (US) reported influenza coverage in 2014/2015 was slightly lower than the Australian estimates for people aged ≥65 years at 66.7 %. Coverage for those aged <65 years with risk factors in the US, was more similar to the Australian coverage reported at 47.6 % [34]. Compared to the United Kingdom (UK) and the US, the pneumococcal vaccination coverage reported from Australian studies among those aged ≥65 years appeared somewhat lower; in 2015 in England coverage was 69.8 % [35] and in the US in 2013 it was 59.7 % [36]. However, these percentages are ‘ever received’ pneumococcal vaccination compared to the Australian coverage presented in this paper which generally reports on receipt of vaccine in the previous 5 years.

There were few studies reporting on factors other than age associated with influenza or pneumococcal vaccination status. Of all the factors reported to be consistently associated with vaccination in this review of Australian data (lower education, lower income, a medical risk factor, being female, living in a metropolitan area, having a positive perception of vaccination, doctor recommending influenza vaccination to the patient, having poor self-reported health status and believing the vaccine to be effective), each has also been found to be significantly associated with influenza vaccination internationally [37–44]. However, some studies from other countries report variable relationships with sex and education [40]. Given the small number of studies, however, more information regarding factors associated with vaccination in Australia would assist in better targeting of vaccination programs. The findings in relation to lower education and lower income have implications for future interventions and further investigation into this issue would be of benefit.

To our knowledge, this is the first systematic review of adult vaccination coverage in Australia. Limitations include that coverage was not always reported in consistent age groups and/or the raw data were not available. This restricted the number of studies that could be included in the meta-analysis. Also, many reports of coverage are found in the grey literature rather than easily searchable electronic databases, which means that there is a possibility that some sources were missed. However, we minimised this by consulting with experts. A majority of studies relied on self-reported data. Self-reported influenza and pneumococcal vaccination status have been shown to be highly sensitive, but less specific [45–47] which may mean that studies using self-report are over-reporting coverage. To date there has been little work carried out to validate provider report or the state-based databases which were used by other studies to obtain coverage estimates although it is likely that these sources would underestimate coverage due to vaccinations occurring in different sites or providers not reporting to the state-based databases. It may be that this difference underlies the heterogeneity in coverage reports we found for pneumococcal vaccination in those ≥65 years. Quantifying the accuracy of different data sources for estimating adult vaccine coverage is an important area for future research.

The higher level of both influenza and pneumococcal vaccination coverage in people aged ≥65 years in Australia since funding for each vaccine was introduced suggests that programs that cover vaccine costs can increase adult vaccination coverage. However, other factors are also likely to play a part, as pneumococcal vaccine coverage after 2005 appeared to be lower than influenza in the ≥65 year age group despite both vaccines being funded. This may be due to the increased complexity of the pneumococcal schedule, and lack of confidence in the vaccine due to limited evidence supporting its effectiveness in adults [48]. In addition, influenza vaccination coverage in people aged <65 years with risk factors, which were also funded after 2010, had much lower vaccination coverage than those in the universally funded group (≥65 years). These findings suggest that whilst funding is important, the way in which funding is provided can also impact. It also highlights the complex nature of adult vaccination and the challenges in obtaining high vaccine coverage in some populations. Lower coverage is often found in targeted programs due to the increased complexity, difficulty in identifying patients and being aware of eligibility [49]. There is some evidence that adult vaccination coverage in funded groups can be increased when combined with other strategies aimed at primary care providers. Some strategies which have shown success include identifying one key staff member in a practice to plan and lead an influenza campaign [50], educational outreach aimed at primary care [50], patient tracking and provider prompts [51].

We found no evidence that Aboriginal and Torres Strait Islander adults aged ≥65 years had substantially different annual influenza coverage than the general population of adults ≥65 years. However, this was based on findings from 3 studies from early last decade [20, 21]. There were also variations in the estimates of coverage provided for Aboriginal and Torres Strait Islander adults, making comparisons difficult. Given the increased vulnerability of Aboriginal and Torres Strait Islander adults to respiratory illnesses such as influenza and pneumococcal disease [52] and specific programs targeting these adults, more current and detailed data on vaccination coverage in this group is warranted.

## Conclusions

We found that although the majority of adults in the general population aged ≥65 years are receiving vaccination for influenza and for pneumococcal diseases, coverage is much lower than that for childhood vaccines. In people aged <65 years with co-morbidities, who are at higher risk of influenza than those without, vaccine coverage is substantially lower than in those aged ≥65 years, despite this group being funded for free vaccines since 2010. This finding supports suggestions [32] that targeted vaccine programs are less effective at achieving high coverage. Official national vaccination coverage targets for the adult population and programmatic measures to improve overall coverage would be useful. Such measures may include incentives and key performance indicators. This review also highlights the limited sources of information currently available in relation to adult vaccination coverage in Australia, especially in relation to Aboriginal and Torres Strait Islander adults. More data are required to provide better estimates of coverage in this higher risk group and also to identify other groups of adults in whom vaccination coverage could be improved. Given the recent announcement by the federal government to implement a whole-of-life immunisation register in Australia, identifying an effective and efficient methodology to collect accurate data on adult immunisation coverage, especially for the subjects of targeted programs, such as Aboriginal and Torres Strait Islander adults and people with medical risk factors, will be an important priority.

## Figures and Tables

**Fig. 4:**
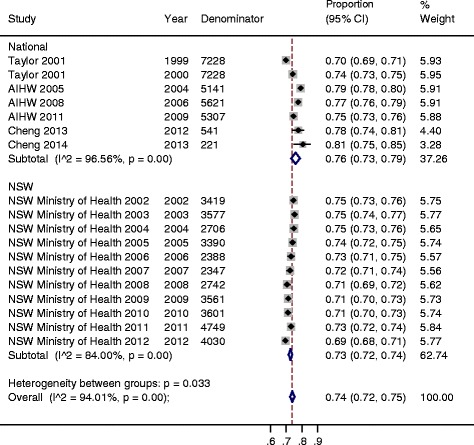
Meta-analysis of eligible studies of influenza vaccination coverage from 1999 for adults aged ≥65 years, by location (National versus NSW). This appendix figure shows the results from the meta-analysis of studies reporting influenza vaccination coverage post introduction of funding by location in people aged ≥65 years

## Abbreviations

ACT: Australian Capital Territory
BEACH: Bettering the evaluation and care of health
CI: Confidence interval
HPV: Human papillomavirus
IQR: Inter quartile range
NHMRC: National Health and Medical Research Council
NIP: National Immunisation Program
NSW: New South Wales
RR: Relative risk
UK: United Kingdom
UNSW: University of New South Wales
US: United States
